# Mechanical Performance Comparison of Sandwich Panels with Graded Lattice and Honeycomb Cores

**DOI:** 10.3390/biomimetics9020096

**Published:** 2024-02-06

**Authors:** Hussam Georges, Diego García Solera, Carlos Aguilar Borasteros, Mohmad Metar, Gyeongseob Song, Rahul Mandava, Wilfried Becker, Christian Mittelstedt

**Affiliations:** 1 Institute for Lightweight Engineering and Structural Mechanics, Technical University of Darmstadt, Otto-Berndt-Straße 2, 64287 Darmstadt, Germany; diego.garcia_solera@stud.tu-darmstadt.de (D.G.S.); carlos.borasteros@stud.tu-darmstadt.de (C.A.B.); akram.metar@stud.tu-darmstadt.de (M.M.); gyeongseob.song@stud.tu-darmstadt.de (G.S.); rahul.mandava@stud.tu-darmstadt.de (R.M.); christian.mittelstedt@lsm.tu-darmstadt.de (C.M.); 2 Institute of Structural Mechanics, Technical University of Darmstadt, Franziska-Braun-Str. 7, 64287 Darmstadt, Germany; becker@fsm.tu-darmstadt.de

**Keywords:** sandwich panels, 3D lattice core, honeycomb core, fully stressed design, graded core, design for additive manufacturing

## Abstract

The design of graded and multifunctional lattice cores is driven by the increasing demand for high-performance components in lightweight engineering. This trend benefits from significant achievements in additive manufacturing, where the lattice core and the face sheets are fabricated simultaneously in a single print job. This work systematically compares the mechanical performance of sandwich panels comprising various graded lattice cores subjected to concentrated loads. In addition to graded lattice cores, uniform lattices and conventional honeycomb cores are analyzed. To obtain an optimized graded lattice core, a fully stressed design method is applied. Stresses and displacements are determined using a linear elastic analytical model that allows grading the core properties in a layerwise manner through the core thickness. The analysis indicates the superior performance of graded lattice cores compared to homogeneous lattice cores. However, conventional honeycombs outperform graded lattice cores in terms of load-to-weight ratio and stiffness-to-weight ratio. This study provides valuable insights for the design of lattice core sandwich panels and the advantages of several design approaches.

## 1. Introduction

In the modern landscape of engineering, sandwich panels have become indispensable in a wide range of disciplines, offering an outstanding combination of strength, lightweight, and energy efficiency [1,2,3]. Typically, these composite structures consist of two thin outer face sheets bonded to an inner lightweight core. Since the core may represent up to more than half of the sandwich weight and the most substantial volume of the structure, an optimal low-density composition is required to achieve an efficient design and save costs [4]. Furthermore, the core is a critical component of these sandwich panels, as it significantly determines their mechanical performance, stiffness, and overall structural integrity [5,6]. Therefore, many studies have investigated novel core designs to improve the efficiency of sandwich panels regarding their strength-to-weight ratio. One of the common approaches to improve the efficiency of the sandwich core is to customize the core properties to the applied load by using graded materials. Compared to conventional homogeneous cores, Jin et al. [7] show that graded honeycomb cores may enhance the dynamic behavior of sandwich panels under blast loading. Furthermore, graded honeycomb cores were investigated by Yu et al. [8]. By varying the cell size and wall thickness of the honeycomb core, the strength and stiffness of the sandwich structure were improved. Analysis of sandwich panels with graded foam cores performed by Conde et al. [9] reveals that the graded core may reduce the structural mass. Besides improving the mechanical performance, graded cores may be used multifunctionally to enhance thermal and acoustic isolation in aerospace applications, as shown by Hohe et al. [10]. Thus, tailoring the core’s properties to save weight and improve the structural performance has never been more relevant, especially in industries where trimming each kilogram of weight leads to significant cost savings and enhanced operational performance [11,12].

An additional approach to improve sandwich panels is using novel materials and structures as one of the keys to weight reduction. For the skin layers, fiber-reinforced composites have replaced the metallic face sheets in numerous applications [13,14]. The performance of sandwich panels depends as well on the topology and material of the core [15]. Several alternative structures may replace honeycomb cores, but these are not used due to a lack of knowledge about the design and mechanical behavior [16,17]. One of the promising structures is additively manufactured lattice materials [18,19]. The integration of additive manufacturing techniques opens new avenues for enhancing both innovation and efficiency in sandwich structures by empowering engineers with the ability to fabricate complex sandwich panels in a single manufacturing step while maintaining precise control over material distribution [20,21,22]. Due to the flexibility in core design through additive manufacturing, Li et al. [23] modified conventional body-centered lattice cores to enhance the structural vibration resistance. Grading the lattice core through the thickness may improve the specific structural stiffness of sandwich panels, as reported by Li et al. [24]. Furthermore, the multifunctional use of the open cell lattice cores as heat exchangers enables an additional lightweight potential of these structures [25,26]. However, lattice cores have a tendency to fail in sandwich panels subjected to concentrated loads due to highly localized stresses in the load application area [27,28]. To reduce the stress concentration and provide a more lightweight core design, the lattice core can be graded through the thickness. Zhang et al. [29] and Boschetto et al. [30] already used additively manufactured graded lattice cores to avoid stress concentrations and enhance the dynamic behavior in satellite housings. Costly and time-consuming finite element analyses are used for the design of such lattice cores, as lattice cores are poorly studied by analytical approaches [31,32]. Therefore, this work introduces an analytical model to determine the lattice core struts’ stresses. The analytical model in this study could simplify and accelerate the design of graded lattice sandwich panels in engineering applications. Moreover, this paper presents a comprehensive analysis of the mechanical performance of sandwich panels subjected to a 3-point bending load with different core configurations: homogeneous lattice and graded lattice. In the graded core, the strut diameter varies in a layerwise manner through the core thickness. Such a stepwise grading approach was used by Song et al. [33] to reduce the computational effort in the modeling. Furthermore, layerwise graded strut-based lattices were investigated by Bai et al. [34] to increase the energy absorption capacity. In this study, the strut diameter of each core layer is determined by using a fully stressed design method (FSD). The number of degrees of freedom within the FSD will be varied so that several graded core designs are compared. As a reference structure for comparison, honeycomb cores are investigated since they have proved extensive application in various industries [35,36]. The comparison between the honeycomb and lattice core is essential to understand the advantages and limitations of each design. By evaluating their mechanical performance under a 3-point bending load, this research aims to provide valuable insights into selecting core structures that are well suited for industrial applications. In addition, it seeks to identify potential areas of improvement in honeycomb and graded lattice cores, pushing the boundaries of structural efficiency and versatility within the framework of sandwich panel technology.

## 2. Theory and Modeling Approach

This section presents the core materials considered in this work and their properties. Furthermore, an energy-based analytical model is derived to determine the struts’ stresses in sandwich panels subjected to a 3-point bending load. Finally, the workflow of the fully stressed design within this study is detailed.

### 2.1. Core Materials

**Figure 1:**
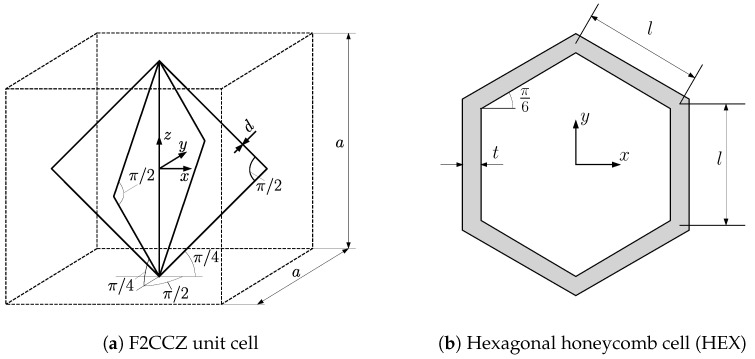
Topology and geometry of the core structures.

Considering the load in a 3-point bending scenario, the core is responsible for transferring the shear stresses and the transverse normal stresses resulting from the localized load. Thus, the selected core material should meet these requirements to withstand the resulting stresses in the core. This study focuses on 3D strut-based lattice structures and regular honeycombs. Souza et al. [37] provided an analytical model based on beam theory to analyze the mechanical performance of numerous strut-based lattice topologies. Based on this model, face-centered lattice cells with vertical struts (F2CCZ) show the highest shear and transverse strength compared to other strut-based lattice materials. Furthermore, this model will be used to obtain the effective properties of the lattice material. Figure 1a illustrates the cubic lattice unit cell of the F2CCZ representative volume element. The cell of size *a* consists of nine struts: four $45^{\circ }$ inclined struts in the xz-plane, four $45^{\circ }$ inclined struts in the yz-plane, and one vertical strut. A uniform diameter *d* is present in all struts. An experimental study performed by Sereshk et al. [38] proved that the mechanical response of the lattice depends merely on the unit cell’s aspect ratio a/d. Thus, the effective stiffness of the lattice changes when the cell size *a* remains constant and the strut diameter *d* varies. Figure 1b shows the regular hexagonal honeycomb cell (HEX). The unit cell’s height and length are assumed to be identical and described by the quantity *l*. According to Gibson and Ashby [39], the in-plane and out-of-plane stiffness of the regular honeycomb can be given as

(1) $$\begin{array}{c} \frac{E_{xx}^{\left(\mathrm{HEX}\right)}}{E_{s}}=\frac{E_{yy}^{\left(\mathrm{HEX}\right)}}{E_{s}}=2.3{\left(\frac{t}{l}\right)}^{3},\;\frac{E_{zz}^{\left(\mathrm{HEX}\right)}}{E_{s}}=1.15\left(\frac{t}{l}\right),\;\frac{G_{xz}^{\left(\mathrm{HEX}\right)}}{G_{s}}=0.57\left(\frac{t}{l}\right), \end{array}$$

and the Poisson’s ratio can be determined by

(2) $$\begin{array}{c} \nu_{xy}^{\left(\mathrm{HEX}\right)}=\frac{\cos^{2}\left(\pi /6\right)}{\sin^{2}\left(\pi /6\right)+\sin\left(\pi /6\right)},\;\mathrm{and}\;\nu_{zx}^{\left(\mathrm{HEX}\right)}=\nu_{zy}^{\left(\mathrm{HEX}\right)}=\nu_{s}, \end{array}$$

where $E_{s}$ and $\nu_{s}$ are the elastic modulus and Poisson’s ratio of the core solid material, respectively.

### 2.2. Sandwich Model

**Figure 2:**
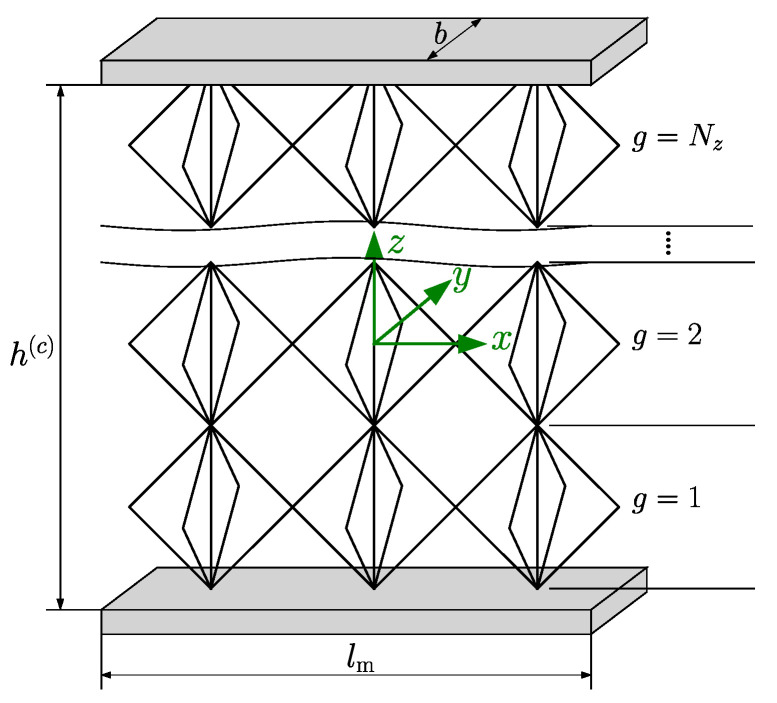
3D sandwich model with lattice core.

In the present study, we examine a sandwich structure exposed to a transverse single point load applied to the midpoint of the upper surface of the top face sheet while being simply supported at the ends of the bottom face sheet. This configuration mirrors the arrangement of a three-point bending test. The number of the lattice unit cells along the sandwich length, through the core thickness and sandwich width, is represented by $N_{x}$, $N_{z}$, and $N_{y}$, respectively. Thus, the length of the sandwich model $l_{m}$, the thickness of the core $h^{\left(c\right)}$, and the width of the sandwich *b* can be determined by $l_{m}=N_{x}\;a$, $h^{\left(c\right)}=N_{z}\;a$, and $b=N_{y}\;a$, as shown in Figure 2.

**Figure 3:**
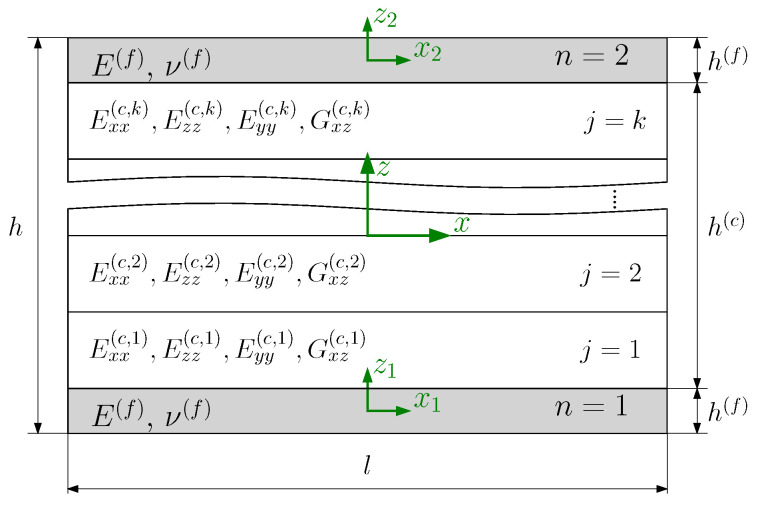
2D sandwich model with homogenized core decomposed into *k* mathematical layers.

In this study, the sandwich thickness is assumed to be negligible in comparison to the sandwich width. Therefore, the 3D sandwich model is reduced to a 2D model, considering only the xz-plane, as illustrated in Figure 3. The modeling approach used in this work is based on the analytical model presented by Georges et al. [40] for the analysis of homogeneous lattice core sandwich panels. The sandwich is decomposed into two face sheets and a core in the analytical model. A Cartesian coordinate system is positioned at the center of each layer. While the face sheets are isotropic with the elastic modulus $E^{\left(f\right)}$ and Poisson’s ratio $\nu^{\left(f\right)}$, a homogenized orthotropic material represents the lattice and honeycomb core using their effective elastic properties. The three sandwich layers are assumed to be ideally bonded. The face sheet and the core exhibit the thickness $h^{\left(f\right)}$ and $h^{\left(c\right)}$, respectively.

Considering a 3-point bending load case, the thin face sheets predominantly transmit the bending stresses. Additionally, shear stresses are anticipated in the skin layers. Therefore, the face sheets show a behavior similar to shear deformable strips. Thus, the horizontal and vertical displacement of the layer mid-axis $u_{0}^{\left(n\right)}$, $w_{0}^{\left(n\right)}$ and the section rotational angle $\psi^{\left(n\right)}$ describe the face sheet deformation. The bottom and top face sheets are indicated by n=1 and n=2, respectively. The face sheet displacement representations are given as

(3) $$\begin{array}{cc} \; & u^{\left(n\right)}\left(x,z_{n}\right)=u_{0}^{\left(n\right)}\left(x\right)+z_{n}\psi^{\left(n\right)}\left(x\right),\;\mathrm{and} \end{array}$$

(4) $$\begin{array}{cc} \; & w^{\left(n\right)}\left(x,z_{n}\right)=w_{0}^{\left(n\right)}\left(x\right), \end{array}$$

which correspond to the displacement field of first-order shear deformation theory (FSDT) [41]. The strains are determined through the derivatives of the deformation functions

(5) $$\begin{array}{cc} \; & \varepsilon_{xx}^{\left(n\right)}=\frac{\partial u_{0}^{\left(n\right)}}{\partial x}+z_{n}\frac{\partial \psi^{\left(n\right)}}{\partial x}, \end{array}$$

(6) $$\begin{array}{cc} \; & \varepsilon_{zz}^{\left(n\right)}=\frac{\partial w^{\left(n\right)}}{\partial z_{n}}=0,\;\mathrm{and} \end{array}$$

(7) $$\begin{array}{cc} \; & \gamma_{xz}^{\left(n\right)}=\frac{\partial w_{0}^{\left(n\right)}}{\partial x}+\psi^{\left(n\right)}. \end{array}$$

Following the assumption of a plane-strain state and isotropic face sheets, the stresses within the face sheet layers can be expressed as follows

(8) $$\begin{array}{cc} \; & \sigma_{xx}^{\left(n\right)}=\frac{E^{\left(f\right)}}{1-\nu^{{\left(f\right)}^{2}}}\varepsilon_{xx}^{\left(n\right)},\;\mathrm{and} \end{array}$$

(9) $$\begin{array}{cc} \; & \tau_{xz}^{\left(n\right)}=G^{\left(f\right)}\gamma_{xz}^{\left(n\right)}. \end{array}$$

The core will be partitioned into *k* mathematical layers to facilitate grading through the core thickness (Figure 3). Each mathematical layer may include several uniform strut diameter lattice layers. As a result, the number of lattice layers may deviate from the number of mathematical core layers, depending on the grading approach. Thus, the total number of lattice physical layers $N_{z}$ is not necessarily identical to that of the mathematical layers *k*. This modeling enables grading the core with low computational effort. The mathematical layer is assigned an effective stiffness $E^{\left(c,j\right)}$ corresponding to the aspect ratio (a/d) of the representative volume element (RVE). The quantity *j* signifies the number of the core’s mathematical layer through the thickness, and *c* indicates the core of the sandwich model. For the homogeneous core, the core will be represented by a single mathematical layer.

**Figure 4:**
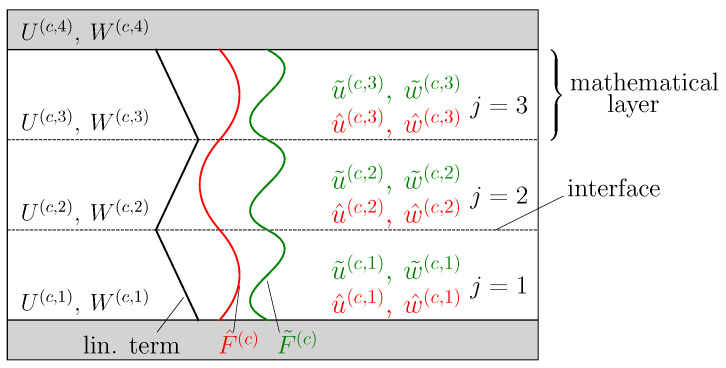
Exemplary core modeling by introducing 3 mathematical layers and higher-order displacement representations.

Unlike the deformation of the face sheet, the intricate behavior of the core deformation necessitates higher-order approaches to ascertain the through-thickness stresses resulting from the local load and the grading. Hence, new degrees of freedom, namely the vertical and horizontal displacements at the interfaces of the mathematical layers, $\left(U^{\left(c,q\right)},W^{\left(c,q\right)}\right)$ are introduced, with *q* being the number of the interface in the sandwich core (Figure 4). The core comprises p=k+1 interfaces, with *q* = 1 and *q* = *p* representing the interfaces between the core and the bottom and top face sheets, respectively. Induced by the stiffness mismatch and concentrated loads, localized deformations with high gradients are anticipated in load and support areas within the core. Hence, higher-order approaches are needed to capture these deformations in an appropriate manner. Consequently, for each core mathematical layer, a linear interpolation term, a quadratic term $\left({\hat{u}}^{\left(c,j\right)},{\hat{w}}^{\left(c,j\right)}\right)$, and a cubic term $\left({\tilde{u}}^{\left(c,j\right)},{\tilde{w}}^{\left(c,j\right)}\right)$ are introduced. Assuming a constant mathematical layer thickness, the mathematical layer displacement representations are chosen as

(10) $$\begin{array}{cc} \; & u^{\left(c,j\right)}\left(x,z\right)={\tilde{f}}_{u}^{\left(c,j\right)}\left(x,z\right)\frac{k(U^{\left(c,j+1\right)}\left(x\right)-U^{\left(c,j\right)}\left(x\right))}{h^{\left(c\right)}}+{\hat{F}}^{\left(c,j\right)}\left(z\right){\hat{u}}^{\left(c,j\right)}\left(x\right)+{\overset{‘}{F}}^{\left(c,j\right)}\left(z\right){\tilde{u}}^{\left(c,j\right)}\left(x\right),\;\mathrm{and} \end{array}$$

(11) $$\begin{array}{cc} \; & w^{\left(c,j\right)}\left(x,z\right)={\tilde{f}}_{w}^{\left(c,j\right)}\left(x,z\right)\frac{k(W^{\left(c,j+1\right)}\left(x\right)-W^{\left(c,j\right)}\left(x\right))}{h^{\left(c\right)}}+{\hat{F}}^{\left(c,j\right)}\left(z\right){\hat{w}}^{\left(c,j\right)}\left(x\right)+{\overset{‘}{F}}^{\left(c,j\right)}\left(z\right){\tilde{w}}^{\left(c,j\right)}\left(x\right). \end{array}$$

The distribution functions ${\tilde{f}}_{u}^{\left(c,j\right)}\left(x,z\right)$, ${\tilde{f}}_{w}^{\left(c,j\right)}\left(x,z\right)$, ${\hat{F}}^{\left(c,j\right)}\left(z\right)$, and ${\tilde{F}}^{\left(c,j\right)}\left(z\right)$ have to respect the displacement continuity conditions at the layer interfaces, ensuring the prevention of displacement discontinuities. Therefore, the functions ${\hat{F}}^{\left(c,j\right)}\left(z\right)$ and ${\tilde{F}}^{\left(c,j\right)}\left(z\right)$ vanish at the interfaces. The expressions for these functions are provided as follows

(12) $$\begin{array}{cc} \; & {\tilde{f}}_{u}^{\left(c,j\right)}\left(x,z\right)=z+\frac{h^{\left(c\right)}}{k(U^{\left(c,j+1\right)}\left(x\right)-U^{\left(c,j\right)}\left(x\right))}\left[\left(k-2j+2\right)U^{\left(c,j+1\right)}\left(x\right)-\left(k-2j\right)U^{\left(c,j\right)}\left(x\right)\right], \end{array}$$

(13) $$\begin{array}{cc} \; & {\tilde{f}}_{w}^{\left(c,j\right)}\left(x,z\right)=z+\frac{h^{\left(c\right)}}{k(W^{\left(c,j+1\right)}\left(x\right)-W^{\left(c,j\right)}\left(x\right))}\left[\left(k-2j+2\right)W^{\left(c,j+1\right)}\left(x\right)-\left(k-2j\right)W^{\left(c,j\right)}\left(x\right)\right], \end{array}$$

(14) $$\begin{array}{cc} \; & {\hat{F}}^{\left(c,j\right)}\left(z\right)=-\left(k-2j+2)(k-2j\right)-\frac{4(k-2j+1)k}{h^{\left(c\right)}}z-\frac{4k^{2}}{h^{{\left(c\right)}^{2}}}z^{2},\;\mathrm{and} \end{array}$$

(15) $$\begin{array}{cc} \; & {\tilde{F}}^{\left(c,j\right)}\left(z\right)={\hat{F}}^{\left(c,j\right)}\left(z\right)\;z\;. \end{array}$$

Decomposing the core into more than 10 layers eliminates the need for third-order terms since the relatively high number of linear and quadratic terms combined with the degrees of freedom at the interfaces can capture the high-order deformations. Therefore, these approaches are not applied when the number of mathematical layers exceeds ten. In contrast, to ensure precise results in the single layer modeling of the homogeneous core, fourth-order terms (${\overset{‘}{F}}^{\left(c,j\right)}\left(z\right)\;{\overset{‘}{u}}^{\left(c,j\right)}\left(x\right)$, ${\overset{‘}{F}}^{\left(c,j\right)}\left(z\right)\;{\overset{‘}{w}}^{\left(c,j\right)}\left(x\right)$) are added to the representations in Equations (10) and (11), where

(16) $$\begin{array}{cc} \; & {\overset{‘}{F}}^{\left(c,j\right)}\left(z\right)={\tilde{F}}^{\left(c,j\right)}\left(z\right)\;z\;. \end{array}$$

Figure 4 shows an example of the core modeling if the core is divided into three mathematical layers. In this case, each mathematical layer has four independent degrees of freedom. In addition, the core has four independent degrees of freedom at the interfaces $\left(U^{\left(c,2\right)},W^{\left(c,2\right)},U^{\left(c,3\right)},W^{\left(c,3\right)}\right)$. The degrees of freedom $\left(U^{\left(c,1\right)},W^{\left(c,1\right)},U^{\left(c,4\right)},W^{\left(c,4\right)}\right)$ depend on the face sheets’ deformation and are not independent

(17) $$\begin{array}{c} u_{0}^{\left(1\right)}\left(x\right)+\frac{h^{\left(f\right)}}{2}\psi^{\left(1\right)}\left(x\right)=U^{\left(c,1\right)}, \end{array}$$

(18) $$\begin{array}{c} w_{0}^{\left(1\right)}\left(x\right)=W^{\left(c,1\right)}, \end{array}$$

(19) $$\begin{array}{c} u_{0}^{\left(2\right)}\left(x\right)-\frac{h^{\left(f\right)}}{2}\psi^{\left(2\right)}\left(x\right)=U^{\left(c,4\right)}, \end{array}$$

(20) $$\begin{array}{c} w_{0}^{\left(2\right)}\left(x\right)=W^{\left(c,4\right)}. \end{array}$$

As each face sheet has three independent degrees of freedom, the total number of degrees of freedom in this case is DOF=22. Separating the core into four and six mathematical layers would increase the degrees of freedom to DOF=28 and DOF=40, respectively.

The derivatives of the displacement functions yield the core strain and are expressed as follows:

(21) $$\begin{array}{cc} \; & \varepsilon_{xx}^{\left(c,j\right)}=\frac{\partial u^{\left(c,j\right)}}{\partial x}, \end{array}$$

(22) $$\begin{array}{cc} \; & \varepsilon_{zz}^{\left(c,j\right)}=\frac{\partial w^{\left(c,j\right)}}{\partial z},\;\mathrm{and} \end{array}$$

(23) $$\begin{array}{cc} \; & \gamma_{xz}^{\left(c,j\right)}=\frac{\partial u^{\left(c,j\right)}}{\partial z}+\frac{\partial w^{\left(c,j\right)}}{\partial x}. \end{array}$$

According to the model’s division into mathematical layers, each mathematical layer may display its own stiffness. Following the assumption of a linear elastic material behavior and a plane-strain state, the core stresses can be derived from the following relationships

(24) $$\begin{array}{c} \left[\begin{array}{c} \sigma_{xx}^{\left(c,j\right)}\\ \sigma_{zz}^{\left(c,j\right)}\\ \tau_{xz}^{\left(c,j\right)} \end{array}\right]\;=\;\left[\begin{array}{ccc} \frac{E_{xx}^{\left(c,j\right)}\left(1-\nu_{zy}^{\left(c,j\right)}\nu_{yz}^{\left(c,j\right)}\right)}{\alpha } & -\frac{E_{xx}^{\left(c,j\right)}\left(\nu_{zx}^{\left(c,j\right)}+\nu_{yx}^{\left(c\right)}\nu_{zy}^{\left(c,j\right)}\right)}{\alpha } & 0\\ -\frac{E_{xx}^{\left(c,j\right)}\left(\nu_{zx}^{\left(c,j\right)}+\nu_{yx}^{\left(c\right)}\nu_{zy}^{\left(c,j\right)}\right)}{\alpha } & \frac{E_{zz}^{\left(c,j\right)}\left(1-\nu_{xy}^{\left(c,j\right)}\nu_{yx}^{\left(c,j\right)}\right)}{\alpha } & 0\\ 0 & 0 & G_{xz}^{\left(c,j\right)} \end{array}\right]\;\left[\begin{array}{c} \varepsilon_{xx}^{\left(c,j\right)}\\ \varepsilon_{zz}^{\left(c,j\right)}\\ \gamma_{xz}^{\left(c,j\right)} \end{array}\right], \end{array}$$

where

(25) $$\begin{array}{c} \alpha =1-\nu_{xy}^{\left(c,j\right)}\left(\nu_{yx}^{\left(c,j\right)}+\nu_{yz}^{\left(c,j\right)}\nu_{zx}^{\left(c,j\right)}\right)-\nu_{yz}^{\left(c,j\right)}\nu_{zy}^{\left(c,j\right)}-\nu_{xz}^{\left(c,j\right)}\left(\nu_{zx}^{\left(c,j\right)}+\nu_{yx}^{\left(c,j\right)}\nu_{zy}^{\left(c,j\right)}\right). \end{array}$$

**Figure 5:**
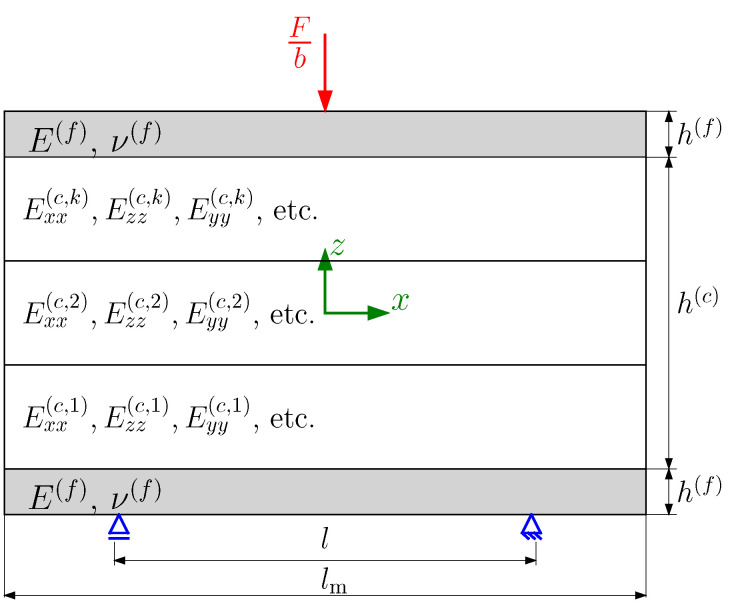
Sandwich model under 3-point bending load.

When considering a 3-point bending problem illustrated in Figure 5, the inner potential energy of the sandwich layers is given as follows

(26) $$\Pi_{i}^{\left(n\right)}=\frac{1}{2}\int_{-l_{m}/2}^{l_{m}/2}\int_{-h^{\left(f\right)}/2}^{h^{\left(f\right)}/2}\left(\sigma_{xx}^{\left(n\right)}\varepsilon_{xx}^{\left(n\right)}+\tau_{xz}^{\left(n\right)}\gamma_{xz}^{\left(n\right)}\right)\;dz\;dx,\;\mathrm{and}$$

(27) $$\Pi_{i}^{\left(c\right)}=\frac{1}{2}\sum_{j=1}^{k}\int_{-l_{m}/2}^{l_{m}/2}\int_{h^{\left(c\right)}\left(2j-k-2\right)/2k}^{h^{\left(c\right)}\left(2j-k\right)/2k}\left(\sigma_{zz}^{\left(c,j\right)}\varepsilon_{zz}^{\left(c,j\right)}+\tau_{xz}^{\left(c,j\right)}\gamma_{xz}^{\left(c,j\right)}+\sigma_{xx}^{\left(c,j\right)}\varepsilon_{xx}^{\left(c,j\right)}\right)\;dz\;dx.$$

According to Figure 5, the following geometric boundary conditions apply

(28) $$w_{0}^{\left(1\right)}\left(x=-\frac{l}{2}\right)=w_{0}^{\left(1\right)}\left(x=\frac{l}{2}\right)=u_{0}^{\left(1\right)}\left(x=\frac{l}{2}\right)=0.$$

The summation of the layers’ inner energies yields the sandwich’s total inner energy

(29) $$\Pi_{i}=\Pi_{i}^{\left(1\right)}+\Pi_{i}^{\left(2\right)}+\Pi_{i}^{\left(c\right)}.$$

With the external potential energy $\Pi_{e}$ resulting from the applied load

(30) $$\begin{array}{cc} \; & \Pi_{e}=\frac{-F}{b}w_{0}^{\left(2\right)}\left(x=0\right), \end{array}$$

the total potential energy is obtained from

(31) $$\begin{array}{cc} \; & \Pi =\Pi_{e}+\Pi_{i}. \end{array}$$

Applying the principle of minimum potential energy with the condition δΠ=0 results in a linear second-order coupled differential equation system

(32) $$\begin{array}{c} \underline{\underline{A}}\;\underline{\ddot{\Psi }}+\underline{\underline{B}}\;\underline{\dot{\Psi }}+\underline{\underline{C}}\;\underline{\Psi }=\underline{0}, \end{array}$$

where $\underline{\Psi }$ includes all deformation functions of the sandwich and $\underline{\dot{\Psi }}$ and $\underline{\ddot{\Psi }}$ are the first and second derivative of $\underline{\Psi }$ with respect to the x-coordinate. The unknown deformation functions are obtained by converting the second-order equation system to a first-order differential equation system

(33) $$\begin{array}{c} \underline{\dot{\Phi }}=\underline{\underline{E}}\;\underline{\Phi }, \end{array}$$

where

(34) $$\begin{array}{cc} \; & \underline{\Phi }={\left[\begin{array}{cc} \underline{\Psi } & \underline{\dot{\Psi }} \end{array}\right]}^{T},\;\;\underline{\underline{E}}=-{\underline{\underline{D_{1}}}}^{-1}\underline{\underline{D_{2}}}, \end{array}$$

(35) $$\begin{array}{cc} \; & \underline{\underline{D_{1}}}=\left[\begin{array}{cc} \underline{\underline{B}} & \underline{\underline{A}}\\ \underline{\underline{I}} & \underline{\underline{0}} \end{array}\right],\;\mathrm{and}\;\;\;\;\underline{\underline{D_{2}}}=\left[\begin{array}{cc} \underline{\underline{C}} & \underline{\underline{0}}\\ \underline{\underline{0}} & -\underline{\underline{I}} \end{array}\right], \end{array}$$

where the matrix $\underline{\underline{I}}$ is a unit matrix. The actual solution for all component functions of the vector $\underline{\Phi }$ results from the eigenvectors ${\underline{v}}_{Q}$ and eigenvalues $\lambda_{Q}$ of the matrix $\underline{\underline{E}}$ as

(36) $$\begin{array}{c} \underline{\Phi }=\sum_{Q=1}^{2DOF}K_{Q}{\underline{v}}_{Q}\;e^{\lambda_{Q}x}\;. \end{array}$$

**Figure 6:**
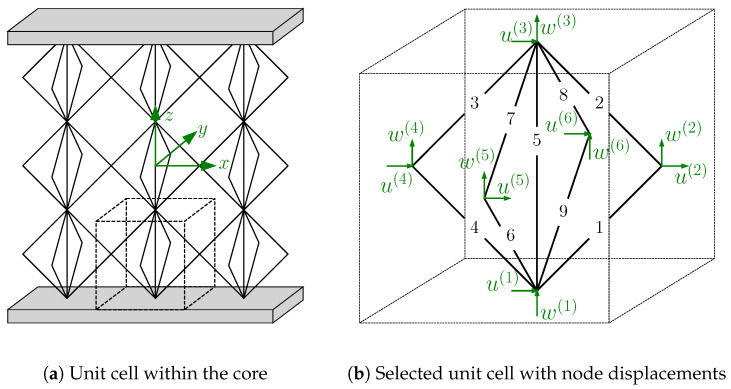
Scheme to determine the struts’ stresses using the node displacements at the lattice nodes.

Although the core is homogenized, the displacements at the lattice node coordinates $u^{\left(m\right)},w^{\left(m\right)}$ are obtained by Equation (36), where *m* is the node’s number in the unit cell, as illustrated in Figure 6b. These displacements are required to determine the lattice struts’ stresses. First, the change in length in each of the struts $\Delta l^{\left(s\right)}$ can be calculated by

(37) $$\begin{array}{cc} \; & \Delta l^{\left(1\right)}=\left(u^{\left(2\right)}-u^{\left(1\right)}\right)\;\cos\left(\pi /4\right)+\left(w^{\left(2\right)}-w^{\left(1\right)}\right)\;\sin\left(\pi /4\right),\\ \; & \Delta l^{\left(2\right)}=\left(u^{\left(3\right)}-u^{\left(2\right)}\right)\;\cos\left(3\pi /4\right)+\left(w^{\left(3\right)}-w^{\left(2\right)}\right)\;\sin\left(3\pi /4\right),\\ \; & \Delta l^{\left(3\right)}=\left(u^{\left(4\right)}-u^{\left(3\right)}\right)\;\cos\left(5\pi /4\right)+\left(w^{\left(4\right)}-w^{\left(3\right)}\right)\;\sin\left(5\pi /4\right),\\ \; & \Delta l^{\left(4\right)}=\left(u^{\left(1\right)}-u^{\left(4\right)}\right)\;\cos\left(-\pi /4\right)+\left(w^{\left(1\right)}-w^{\left(4\right)}\right)\;\sin\left(-\pi /4\right),\\ \; & \Delta l^{\left(5\right)}=\left(w^{\left(3\right)}-w^{\left(1\right)}\right),\\ \; & \Delta l^{\left(6\right)}=\left(w^{\left(5\right)}-w^{\left(1\right)}\right)\;\sin\left(\pi /4\right),\;\;\Delta l^{\left(7\right)}=\left(w^{\left(3\right)}-w^{\left(5\right)}\right)\;\sin\left(\pi /4\right),\\ \; & \Delta l^{\left(8\right)}=\left(w^{\left(6\right)}-w^{\left(3\right)}\right)\;\sin\left(-\pi /4\right),\;\mathrm{and}\;\;\;\Delta l^{\left(9\right)}=\left(w^{\left(1\right)}-w^{\left(6\right)}\right)\;\sin\left(-\pi /4\right). \end{array}$$

Assuming that the lattice joints transfer only forces, the stress in the *s*-th lattice strut results from the change in the length of the *s*-th lattice strut, the strut’s length $l^{\left(s\right)}$, and the elastic modulus $E^{\left(s\right)}$ as

(38) $$\begin{array}{c} \sigma^{\left(s\right)}=E^{\left(s\right)}\;\frac{\Delta l^{\left(s\right)}}{l^{\left(s\right)}}. \end{array}$$

### 2.3. Fully Stressed Design

**Figure 7:**
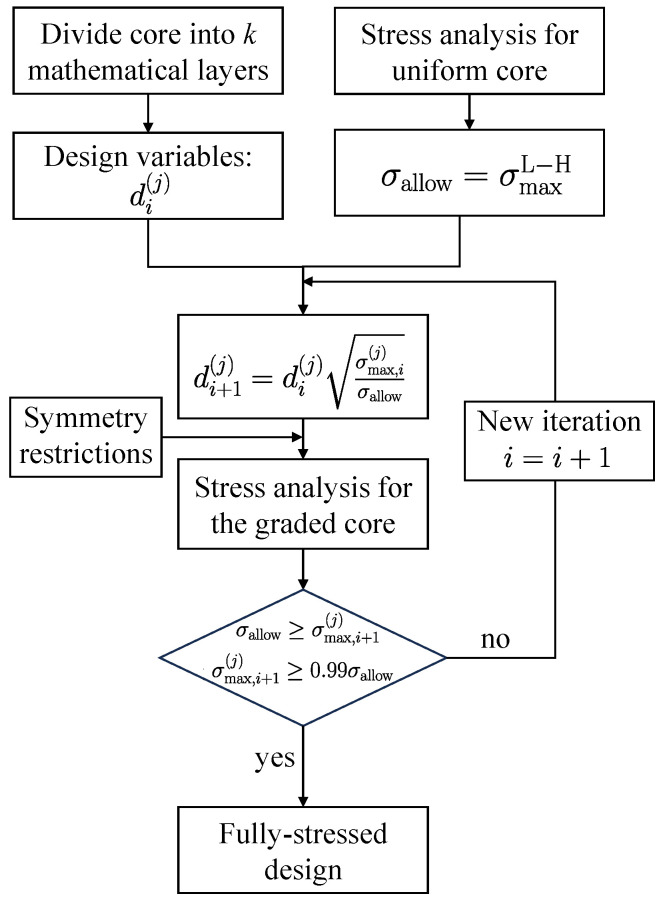
Flowchart representing the steps to determine the fully stressed design of the lattice core.

Fully stressed design (FSD) is a size optimization method that belongs to the so-called heuristic methods. Its criterion is based on reducing the thickness of the lattice struts so that the struts are fully stressed under the given loading conditions. This means that material is removed from those members of the lattice that are not fully loaded, unless a minimum thickness limits them. As a heuristic method, it cannot always guarantee the optimum solution, but it provides a sufficient approximation in many cases. Hence, it is practical and effective. Furthermore, FSD is typically one of the most straightforward but efficient ways to optimize these truss structures [42]. The computational cost is less than the cost of using more complex optimization methods to ensure an optimal solution [43]. Figure 7 shows the FSD scheme used in this study, which can be summarized in the following steps:

1. The core is divided into *k* mathematical layers. The strut diameter of each mathematical layer $d^{\left(j\right)}$ represents a design variable.
2. The struts’ stresses are determined first in the homogeneous lattice core (iteration i=1). The maximum stress found in the homogeneous core is set to the allowable stress limit $\sigma_{\mathrm{allow}}=\sigma_{\max}^{\left(L-H\right)}$.
3. The strut diameter of the *j*-th mathematical layer for the next iteration i+1 is calculated in the following manner
  (39) $$d_{i+1}^{\left(j\right)}=d_{i}^{\left(j\right)}\sqrt{\frac{\sigma_{\max,i}^{\left(j\right)}}{\sigma_{\mathrm{allow}}}},$$
  where $\sigma_{\max,i}^{\left(j\right)}$ denotes the maximum struts’ stress within the *j*-th mathematical layer.
4. Geometric restrictions are applied to provide a symmetric strut diameter variation through the core thickness.
5. The analysis will be stopped when the following condition $\sigma_{\mathrm{allow}}\geq \sigma_{\max,i+1}^{\left(j\right)}\geq 0.99\sigma_{\mathrm{allow}}$ is statisfied.

### 2.4. Failure Load

The following stress analysis is performed within the assumptions of linear elasticity, and the core is considered as failed when the yield strength of the material $R_{p}$ is reached. To evaluate the strength of the lattice core, the von Mises criterion is used. Thus, the failure load is determined by

(40) $$\begin{array}{c} F_{\mathrm{fail},p}^{\left(L\right)}=F\frac{R_{p}}{\sigma_{\max}^{\left(L\right)}}, \end{array}$$

where $\sigma_{\max}^{\left(L\right)}$ is the maximum stress in the lattice core while applying the load *F*. As the lattice struts are subjected to compressive loads, the critical buckling load should not be exceeded. The failure load due to buckling can be given as

(41) $$\begin{array}{c} F_{\mathrm{fail},\mathrm{buckling}}=F\frac{\sigma_{\mathrm{buckling}}^{\left(L\right)}}{\sigma_{\max}^{\left(L\right)}}, \end{array}$$

where the critical buckling stress $\sigma_{\mathrm{buckling}}^{\left(L\right)}$ is obtained using the conservative assumption of simply supported ends of the lattice struts [44,45]. The critical buckling strength results as

(42) $$\begin{array}{c} \sigma_{\mathrm{buckling}}^{\left(L\right)}=\frac{\pi^{2}E_{s}}{16}{\left(\frac{d}{a}\right)}^{2}. \end{array}$$

In contrast to the lattice core, effective stresses are determined for the honeycomb core, as the honeycomb is replaced by an orthotropic homogeneous material using the effective stiffness of the honeycomb core. Following [39], the effective yield strength of regular honeycombs can be determined by

(43) $$\begin{array}{c} \sigma_{p}^{\left(\mathrm{HEX}\right)}=1.15\left(\frac{t}{l}\right)R_{p}. \end{array}$$

The failure load results in

(44) $$\begin{array}{c} F_{\mathrm{fail},p}^{\left(\mathrm{HEX}\right)}=F\frac{\sigma_{p}^{\left(\mathrm{HEX}\right)}}{\sigma_{\max}^{\left(\mathrm{HEX}\right)}}. \end{array}$$

Since the compressive stresses induced by the localized load are the dominant stress component, the honeycomb walls may buckle before the yield strength is reached [39]. The critical buckling load for regular honeycombs can be given as

(45) $$\begin{array}{c} \sigma_{\mathrm{buckling}}^{\left(\mathrm{HEX}\right)}=5.26{\left(\frac{t}{l}\right)}^{3}E_{s}. \end{array}$$

## 3. Results and Discussion

**Figure 8:**
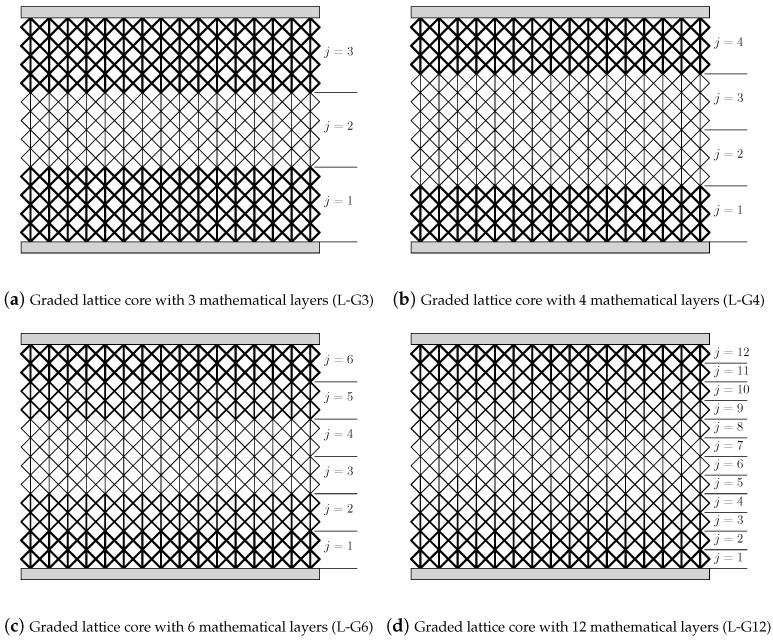
Sandwich panels with graded lattice cores using different grading aprroaches.

In this study, the analysis of sandwich panels with fully stressed designed F2CCZ core is conducted. The load case is 3-point bending, where the top face sheet is subjected to a concentrated load at the center of the upper surface, and the sandwich is simply supported at the lower edges. The core of the sandwich panel is made of 12 lattice physical layers ($N_{z}=12$), and the strut diameter of the cells varies symmetrically and layerwise through the core thickness. In total, four fully stressed designed cores are investigated, as shown in Figure 8. In the first core design (Figure 8a), the core is separated into three mathematical layers that consist of four lattice layers, respectively. The strut diameter within the mathematical layer remains constant. Due to the symmetric distribution of the strut diameter through the core thickness, the three strut diameters are reduced to two design variables. This core design with the graded lattice core separated into three mathematical layers is abbreviated as L-G3 in the following. In the second core design (Figure 8b), four mathematical layers are introduced to the core so that three lattice layers build one mathematical layer (L-G4). Although four strut diameters are available in this design, the number of design variables remains at two due to the symmetry restriction. In the third core design (Figure 8c), the number of the core mathematical layers increases to six (L-G6). In contrast to the first and the second designs, three available design variables exist. In the fourth design (Figure 8d), each mathematical layer represents a lattice physical layer. Due to the symmetry restriction, the number of design variants increases to 6, even though 12 strut diameters are available in this design. To highlight the benefits of grading the core, a sandwich structure with an F2CCZ core and uniform strut diameter (L-H) and a sandwich structure with a regular hexagonal honeycomb core (HEX) are considered in this study. The maximum stress in the homogeneous F2CCZ core is used as the stress limit for the fully stressed design method. The aspect ratio of the homogeneous F2CCZ core is a/d=10. The honeycomb cell wall thickness *t* and length *l* are selected so that the homogeneous F2CCZ core and the honeycomb core exhibit the same relative density. The geometric dimensions of the investigated sandwich panel are based on the sandwich structures manufactured and tested by Kotzem et al. [27], Hao et al. [46]. Thus, the core thickness is set to $h^{\left(c\right)}=$ 36 mm, and the core thickness to face sheet thickness is $h^{\left(c\right)}/h^{\left(f\right)}=$ 12. The length-to-thickness ratio is $l_{m}/h^{\left(c\right)}=8$. The materials of the lattice struts and the face sheets are assumed to be made of an aluminium alloy with the elastic modulus $E_{s}=70$ GPa, the Poisson’s ratio $\nu_{s}=0.35$, and the yield strength $R_{p}=250$ MPa. The honeycomb core is made of the same material as the lattice core.

**Figure 9:**
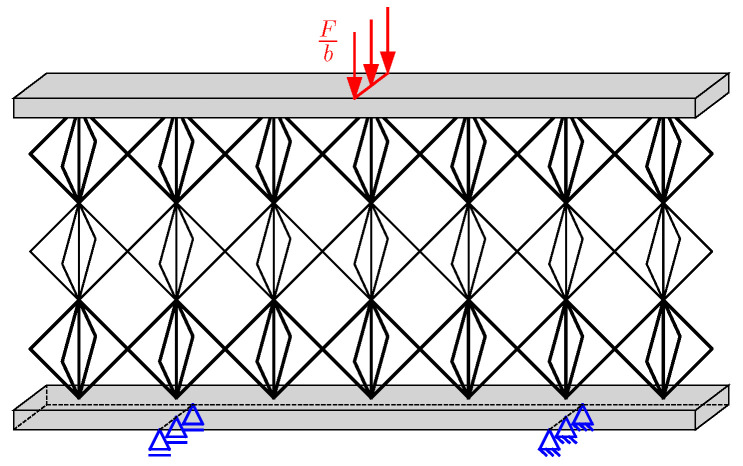
Exemplary illustration of the load application and boundary conditions in the 3D FE model of the lattice core sandwich.

**Figure 10:**
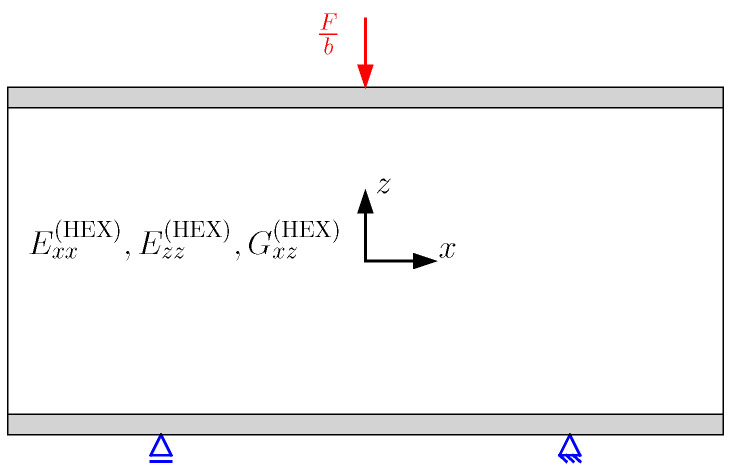
Load application and boundary conditions in the 2D FE model of the sandwich with the homogenized honeycomb core.

To verify the results determined by the analytical model, finite element analysis (FEA) is performed for the sandwich with the homogeneous lattice core, the honeycomb core, and the final version of each fully stressed graded core design. The FEA conducted in ABAQUS CAE Software uses 3D solid elements to model the face sheets and Timoshenko beam elements for the lattice struts. The struts are bonded with the face sheets using tie constraints. The mesh size is $h^{\left(f\right)}/5$. The core consists of one unit cell in the *y*-direction ($N_{y}=1$). To match the plane-strain state assumption, the displacements in *y*-direction are suppressed in the finite element model [47]. The load is applied as a line load on the upper face sheet. An exemplary finite element model is shown in Figure 9. In the FE model of the sandwich with the honeycomb core, the core is modeled as a contiuum core since the effective properties represent the honeycomb structure. Thus, a 2D FE-model using plane-strain continuum elements with the corresponding boundary conditions is created, as shown in Figure 10. The load in the 2D model is applied as a concentrated load.

**Figure 11:**
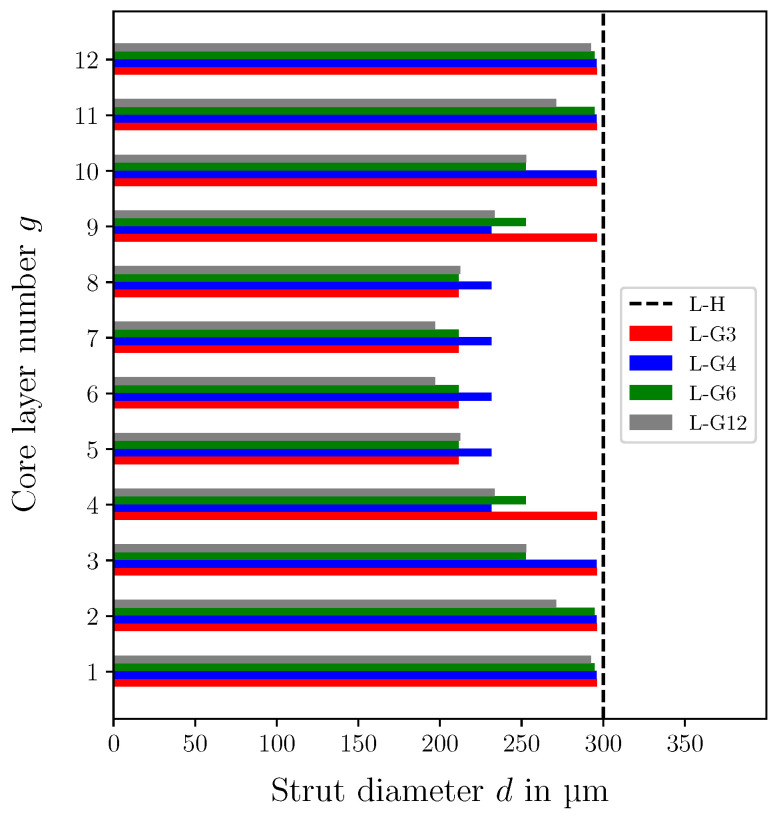
Strut diameters of the 12 core physical layers in the graded lattice cores determined by the fully stressed design and the uniform lattice core. (L-H): lattice core with unifrom strut diameter, (L-G3): graded lattice core with 3 mathematical layers, (L-G4): graded lattice core with 4 mathematical layers, (L-G6): graded lattice core with 6 mathematical layers, (L-G12): graded lattice core with 12 mathematical layers.

In Figure 11, the strut diameters of the core physical layers for all fully stressed lattice core designs are illustrated. All cores have nearly the same strut diameter in the top and bottom layers. However, each design has a different distribution depending on the grading approach selected and the number of design variables. Since the core structure is statically indeterminate, several iterations are required to determine the final design. In this study, approx. 4 iterations were required to reach the fully stressed design. Due to the core grading, the mass of the sandwich was reduced depending on the grading approach between 22% and 46% compared to the sandwich with the uniform core.

**Figure 12:**
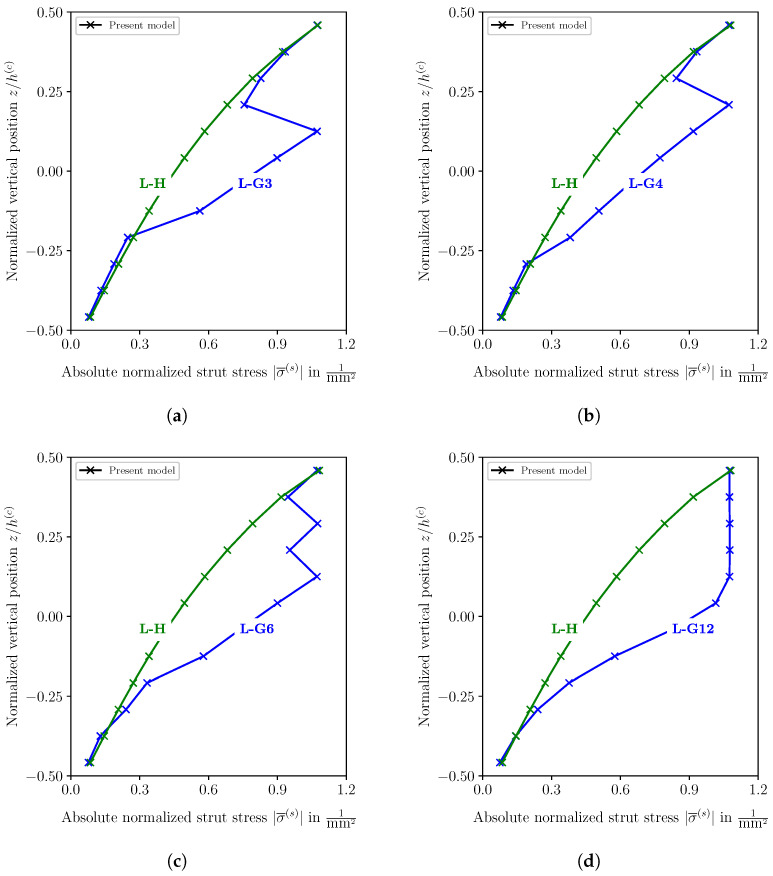
Absolute stress in vertical struts of the lattice core through the core thickness at the center of the sandwich (x=0) for several graded cores and homogeneous cores. (**a**) Homogeneous lattice core (L-H) and graded lattice core with 3 mathematical layers (L-G3), (**b**) homogeneous lattice core (L-H) and graded lattice core with 4 mathematical layers (L-G4), (**c**) homogeneous lattice core (L-H) and graded lattice core with 6 mathematical layers (L-G6), (**d**) homogeneous lattice core (L-H) and graded lattice core with 12 mathematical layers (L-G12).

Figure 12 shows the normalized struts’ stresses (${\bar{\sigma }}^{\left(s\right)}=\sigma^{\left(s\right)}/F$) at the load application point through the core thickness of the graded and homogeneous cores. Due to the concentrated load, the vertical struts at the center of the sandwich are the highest-loaded struts of the lattice core. While the stress variation in L-G3, L-G4, and L-G6 exhibits a zig-zag shape, the stress shows a homogeneous shape through the upper core half of the L-G12 design and decreases to a minimum value at the bottom edge of the core. This variation of the stress in L-G12 is due to the layerwise change of the strut diameter through the core thickness, as each layer represents a core physical layer. In contrast, the mathematical layers in L-G3, L-G4, and L-G6 involve numerous lattice physical layers. The stress distribution in the homogeneous core is given for comparison in all diagrams in Figure 12. It can be seen that the struts’ stresses in the graded cores do not exceed the maximum stress of the homogeneous core, which is the condition of the fully stressed design.

**Figure 13:**
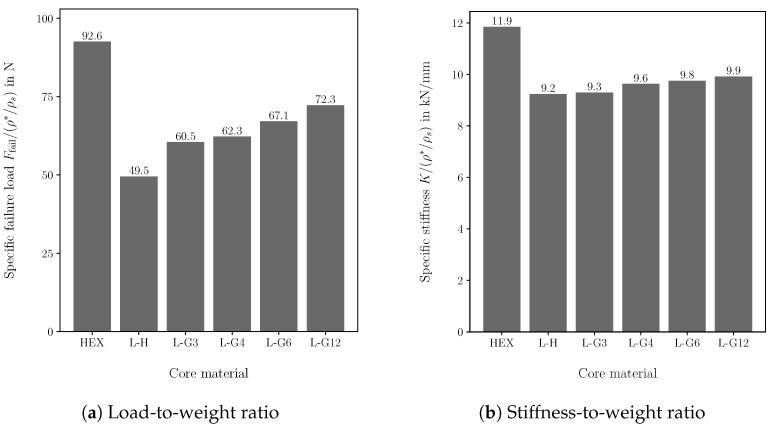
Specific mechanical performance of sandwich panels with different core materials. (HEX): honeycomb core, (L-H): lattice core with unifrom strut diameter, (L-G3): graded lattice core with 3 mathematical layers, (L-G4): graded lattice core with 4 mathematical layers, (L-G6): graded lattice core with 6 mathematical layers, (L-G12): graded lattice core with 12 mathematical layers.

For the performance comparison, the von Mises strength criterion and Euler buckling criterion are used to evaluate the strength and stability of the lattice cores. The failure load $F_{\mathrm{fail}}$ is divided by the core’s relative density to evaluate the sandwich structure’s load-to-weight ratio. Figure 13a illustrates the comparison of the specific failure load for the different core materials. By varying the strut diameter, the failure load of the graded cores increases up to 46% compared to the homogeneous lattice core. The load-to-weight ratio increases mainly as the number of design variables defined in the fully loaded design increases. Thus, the L-G12 design provides the highest increase in specific load capacity, and the L-G3 design provides the lowest increase, which is only approx. 22% compared to the homogeneous lattice core (L-H). However, the honeycomb core outperforms all lattice cores. The load-to-weight ratio of the honeycomb core is up to 28% higher than that of the lattice core with the highest specific failure load (L-G12). Thus, it has been demonstrated that grading the core may significantly improve the mechanical performance of the sandwich panel. In addition to the load-to-weight ratio, the stiffness-to-weight ratio is used to assess the specific stiffness of the sandwich panel. The slope of the force–displacement curve *K* is used to determine the stiffness-to-weight ratio. The displacement is evaluated at the middle of the bottom face sheet. Again, the sandwich with the honeycomb core shows an at least 19% higher specific stiffness than the lattice core designs, as shown in Figure 13b. Although the deflection of the sandwich increases due to the core grading, the specific stiffness increases since the core weight decreases. The increase in the specific stiffness due to the core grading is up to 7% compared to the homogeneous lattice core. As the graded design was stress driven, the increase in the specific failure load is higher than the increase in the specific stiffness. The outcomes of comparing honeycomb core and the uniform lattice core match with the experimental results for sandwich panels with lattice and honeycomb cores presented by Monteiro et al. [48].

**Figure 14:**
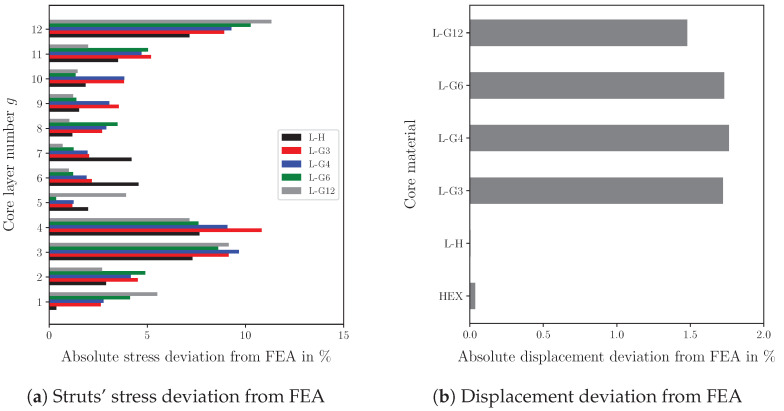
Deviation of the results determined by the analytical model from the finite element analyis.

It is worth noting that the stiffness of the sandwich depends on its length-to-thickness ratio. Neglecting the core indentation and assuming a high axial stiffness of the face sheets compared to the core stiffness, the total deflection of the sandwich consists of two components [49]. The first component $w_{\mathrm{bending}}$ is induced by the resulting bending moment, and it depends mainly on the stiffness of the face sheets ($w_{\mathrm{bending}}=Fl^{3}/\left(48E^{\left(f\right)}I_{yy}\right)$ with $I_{yy}$ the face sheets’ second moment of inertia with respect to the center of gravity of the sandwich). The second component $w_{\mathrm{shear}}$ results from the shear load and decreases with increasing shear stiffness ($w_{\mathrm{shear}}=Fl/\left(4G_{xz}^{\left(c\right)}A\right)$ with *A* the effective shear area of the sandwich). It can be recognized that in thinner sandwich panels, the bending component $w_{\mathrm{bending}}$ will be the dominant term in the total deflection, and a decrease in the core’s shear stiffness will not significantly increase the total deflection of the sandwich. If the length is tripled, the bending component would increase by a factor of 27, while the shear component would merely triple. Therefore, the stiffness of the sandwich is affected by the sandwich’s length. However, the strength of the core is independent of the length, as we observed in previous studies [50]. To verify the results determined by the derived model, the deviation from the results obtained by the finite element model is determined. Figure 14 shows the deviation of the calculated stresses and deflections from the FEA solution. Since the analytical model uses a displacement-based approach, the deflections are rather accurately determined by the derived model, particularly for the homogeneous lattice and honeycomb core. Compared to the deflection results, the prediction of the struts’ stresses is less precise, but it remains reasonable. The deviation for calculating the honeycomb core’s critical stress is merely 2.67%. While the FEA requires approx. five minutes to calculate the struts’ stresses, the model presented provides the results in less than one minute for the uniform core.

## 4. Summary and Conclusions

Thanks to advances in additive manufacturing, the fabrication of graded lattices is enabled. These novel structures can be used as cores in sandwich panels to improve their mechanical performance. This study compared the performance of sandwich panels with several core materials. Using fully stressed design, four lattice core designs with variable strut diameters through the core thickness were analyzed and compared to uniform lattice cores and honeycomb cores. An analytical model was derived to determine the stresses and deflections in the sandwich panels. Compared to the finite element method, the analytical approach presented in this study provides an efficient method to obtain stresses and displacements in graded and homogeneous sandwich cores. The performance comparison between the core materials demonstrated that grading the core may enhance the load-to-weight ratio up to 46% compared to homogeneous lattice core. Furthermore, the specific stiffness was improved by up to 7%. While the graded lattice cores show higher specific strength and stiffness than the homogeneous lattice core, the sandwich with the conventional honeycomb core outperforms the graded lattice core sandwiches in terms of specific strength and stiffness. Although the lattice core sandwich panels show lower performance, the lattice cores are very relevant for applications requiring high impact resistance, energy absorption, or multifunctional use of sandwich structures [51,52,53,54,55]. The derived model can be used to determine stresses and deformations in those cases. The outcomes are expected to provide guidance for core structure choices in the future, helping engineers and researchers in decision making when developing advanced structures and lightweight components.

## Data Availability

The processed data required to reproduce these findings will be provided by the authors upon request.

## References

[1] Fatt M.S.H., Sirivolu D. (2017). Marine composite sandwich plates under air and water blasts. Mar. Struct..

[2] Zinno A., Fusco E., Prota A., Manfredi G. (2010). Multiscale approach for the design of composite sandwich structures for train application. Compos. Struct..

[3] Chai G.B., Zhu S. (2011). A review of low-velocity impact on sandwich structures. Proc. Inst. Mech. Eng. Part L J. Mater. Des. Appl..

[4] He M., Hu W. (2008). A study on composite honeycomb sandwich panel structure. Mater. Des..

[5] Ma Q., Rejab M., Siregar J., Guan Z. (2021). A review of the recent trends on core structures and impact response of sandwich panels. J. Compos. Mater..

[6] Manalo A., Aravinthan T., Fam A., Benmokrane B. (2017). State-of-the-art review on FRP sandwich systems for lightweight civil infrastructure. J. Compos. Constr..

[7] Jin X., Wang Z., Ning J., Xiao G., Liu E., Shu X. (2016). Dynamic response of sandwich structures with graded auxetic honeycomb cores under blast loading. Compos. Part B Eng..

[8] Yu B., Han B., Su P.B., Ni C.Y., Zhang Q.C., Lu T.J. (2016). Graded square honeycomb as sandwich core for enhanced mechanical performance. Mater. Des..

[9] Conde Y., Pollien A., Mortensen A. (2006). Functional grading of metal foam cores for yield-limited lightweight sandwich beams. Scr. Mater..

[10] Hohe J., Hardenacke V., Fascio V., Girard Y., Baumeister J., Stöbener K., Weise J., Lehmhus D., Pattofatto S., Zeng H. (2012). Numerical and experimental design of graded cellular sandwich cores for multi-functional aerospace applications. Mater. Des..

[11] He S.Y., Zhang Y., Dai G., Jiang J.Q. (2014). Preparation of density-graded aluminum foam. Mater. Sci. Eng. A.

[12] Pflug J., Vangrimde B., Verpoest I. (1999). Material efficiency and cost effectiveness of sandwich materials. Proceedings of the International SAMPE Symposium and Exhibition.

[13] Castanie B., Bouvet C., Ginot M. (2020). Review of composite sandwich structure in aeronautic applications. Compos. Part C Open Access.

[14] Chatterjee A., Mishra A., Sharma S., Bhagchandani R.K. (2022). Review on lightweight materials, additive manufacturing techniques and design optimization of an airplane. Proceedings of the AIP Conference Proceedings.

[15] Sugiyama K., Matsuzaki R., Ueda M., Todoroki A., Hirano Y. (2018). 3D printing of composite sandwich structures using continuous carbon fiber and fiber tension. Compos. Part A Appl. Sci. Manuf..

[16] Alomar Z., Concli F. (2020). A review of the selective laser melting lattice structures and their numerical models. Adv. Eng. Mater..

[17] Sun Y., Guo L.C., Wang T.S., Yao L.J., Sun X.Y. (2019). Bending strength and failure of single-layer and double-layer sandwich structure with graded truss core. Compos. Struct..

[18] Austermann J., Redmann A.J., Dahmen V., Quintanilla A.L., Mecham S.J., Osswald T.A. (2019). Fiber-reinforced composite sandwich structures by co-curing with additive manufactured epoxy lattices. J. Compos. Sci..

[19] Mesto T., Sleiman M., Khalil K., Alfayad S., Jacquemin F. (2023). Analyzing sandwich panel with new proposed core for bending and compression resistance. Proc. Inst. Mech. Eng. Part L J. Mater. Des. Appl..

[20] Zaharia S.M., Enescu L.A., Pop M.A. (2020). Mechanical performances of lightweight sandwich structures produced by material extrusion-based additive manufacturing. Polymers.

[21] Khoshgoftar M., Barkhordari A., Limuti M., Buccino F., Vergani L., Mirzaali M. (2022). Bending analysis of sandwich panel composite with a re-entrant lattice core using zig-zag theory. Sci. Rep..

[22] Wang Y., Liu F., Zhang X., Zhang K., Wang X., Gan D., Yang B. (2021). Cell-size graded sandwich enhances additive manufacturing fidelity and energy absorption. Int. J. Mech. Sci..

[23] Li H., Hu Y., Chen J., Shou D., Li B. (2022). Lightweight meta-lattice sandwich panels for remarkable vibration mitigation: Analytical prediction, numerical analysis and experimental validations. Compos. Part A Appl. Sci. Manuf..

[24] Li C., Shen H.S., Wang H., Yu Z. (2020). Large amplitude vibration of sandwich plates with functionally graded auxetic 3D lattice core. Int. J. Mech. Sci..

[25] Bici M., Brischetto S., Campana F., Ferro C.G., Seclì C., Varetti S., Maggiore P., Mazza A. (2018). Development of a multifunctional panel for aerospace use through SLM additive manufacturing. Procedia CIRP.

[26] Tian J., Lu T., Hodson H., Queheillalt D., Wadley H. (2007). Cross flow heat exchange of textile cellular metal core sandwich panels. Int. J. Heat Mass Transf..

[27] Kotzem D., Tazerout D., Arold T., Niendorf T., Walther F. (2021). Failure mode map for E-PBF manufactured Ti6Al4V sandwich panels. Eng. Fail. Anal..

[28] Ghannadpour S., Mahmoudi M., Nedjad K.H. (2022). Structural behavior of 3D-printed sandwich beams with strut-based lattice core: Experimental and numerical study. Compos. Struct..

[29] Zhang X., Zhou H., Shi W., Zeng F., Zeng H., Chen G. (2018). Vibration tests of 3D printed satellite structure made of lattice sandwich panels. AIAA J..

[30] Boschetto A., Bottini L., Macera L., Vatanparast S. (2023). Additive Manufacturing for Lightweighting Satellite Platform. Appl. Sci..

[31] Namasivayam U.M., Seepersad C.C. (2011). Topology design and freeform fabrication of deployable structures with lattice skins. Rapid Prototyp. J..

[32] Karttunen A.T., Reddy J., Romanoff J. (2019). Two-scale micropolar plate model for web-core sandwich panels. Int. J. Solids Struct..

[33] Song J., Tang Q., Feng Q., Ma S., Guo F., Han Q. (2021). Investigation on the modelling approach for variable-density lattice structures fabricated using selective laser melting. Mater. Des..

[34] Bai L., Gong C., Chen X., Sun Y., Xin L., Pu H., Peng Y., Luo J. (2020). Mechanical properties and energy absorption capabilities of functionally graded lattice structures: Experiments and simulations. Int. J. Mech. Sci..

[35] Khan M., Syed A., Ijaz H., Shah R. (2018). Experimental and numerical analysis of flexural and impact behaviour of glass/pp sandwich panel for automotive structural applications. Adv. Compos. Mater..

[36] Suzuki T., Aoki T., Ogasawara T., Fujita K. (2017). Nonablative lightweight thermal protection system for Mars Aeroflyby Sample collection mission. Acta Astronaut..

[37] Souza J., Großmann A., Mittelstedt C. (2018). Micromechanical analysis of the effective properties of lattice structures in additive manufacturing. Addit. Manuf..

[38] Sereshk M.R.V., Triplett K., St John C., Martin K., Gorin S., Avery A., Byer E., St Pierre C., Soltani-Tehrani A., Shamsaei N. (2019). A Computational and Experimental Investigation into Mechanical Characterizations of Strut-Based Lattice Structures. Proceedings of the 2019 International Solid Freeform Fabrication Symposium.

[39] Gibson L.J., Ashby M.F. (1997). The mechanics of honeycombs. Cellular Solids: Structure and Properties.

[40] Georges H., Mittelstedt C., Becker W. (2023). Energy-based strut stress analysis of 3D lattice cores in sandwich panels. Eur. J. Mech. A/Solids.

[41] Reddy J.N. (2003). Mechanics of Laminated Composite Plates and Shells: Theory and Analysis.

[42] Topping B. (1983). Shape optimization of skeletal structures: A review. J. Struct. Eng..

[43] Patnaik S.N., Hopkins D.A. (1998). Optimality of a fully stressed design. Comput. Methods Appl. Mech. Eng..

[44] Zhang L., Chen Y., He R., Bai X., Zhang K., Ai S., Yang Y., Fang D. (2020). Bending behavior of lightweight C/SiC pyramidal lattice core sandwich panels. Int. J. Mech. Sci..

[45] Yuan W., Song H., Huang C. (2016). Failure maps and optimal design of metallic sandwich panels with truss cores subjected to thermal loading. Int. J. Mech. Sci..

[46] Hao N., Wang Y., Song Y., Ruan S., Ma Q., Wang Z. (2023). Load-bearing behaviors of sandwich plates with non-uniformly distributed grid cores: Static compression and bending. J. Mater. Sci..

[47] Omachi A., Ushijima K., Chen D.H., Cantwell W.J. (2020). Prediction of failure modes and peak loads in lattice sandwich panels under three-point loading. J. Sandw. Struct. Mater..

[48] Monteiro J., Sardinha M., Alves F., Ribeiro A., Reis L., Deus A., Leite M., Vaz M.F. (2021). Evaluation of the effect of core lattice topology on the properties of sandwich panels produced by additive manufacturing. Proc. Inst. Mech. Eng. Part L J. Mater. Des. Appl..

[49] Pollien A., Conde Y., Pambaguian L., Mortensen A. (2005). Graded open-cell aluminium foam core sandwich beams. Mater. Sci. Eng. A.

[50] Georges H., Großmann A., Mittelstedt C., Becker W. (2022). Structural modeling of sandwich panels with additively manufactured strut-based lattice cores. Addit. Manuf..

[51] Cui X., Zhao L., Wang Z., Zhao H., Fang D. (2012). Dynamic response of metallic lattice sandwich structures to impulsive loading. Int. J. Impact Eng..

[52] Wang X., Wei K., Wang K., Yang X., Qu Z., Fang D. (2020). Effective thermal conductivity and heat transfer characteristics for a series of lightweight lattice core sandwich panels. Appl. Therm. Eng..

[53] Yan H., Yang X., Lu T., Xie G. (2017). Convective heat transfer in a lightweight multifunctional sandwich panel with X-type metallic lattice core. Appl. Therm. Eng..

[54] Wicks N., Hutchinson J.W. (2004). Performance of sandwich plates with truss cores. Mech. Mater..

[55] Tarlochan F. (2021). Sandwich structures for energy absorption applications: A review. Materials.

